# Microplastics in aquaculture - Potential impacts on inflammatory processes in Nile tilapia

**DOI:** 10.1016/j.heliyon.2024.e30403

**Published:** 2024-04-30

**Authors:** Azora König Kardgar, Darragh Doyle, Niklas Warwas, Terese Hjelleset, Henrik Sundh, Bethanie Carney Almroth

**Affiliations:** Department of Biological and Environmental Sciences, University of Gothenburg, Gothenburg, Sweden

**Keywords:** Plastics pollution, Conventional polymers, Fish farming, *Oreochromis niloticus*, Gut health, Intestine

## Abstract

Aquaculture is essential for meeting the growing global demand for fish consumption. However, the widespread use of plastic and the presence of microplastics in aquaculture systems raise concerns about their impact on fish health and the safety of aquaculture products. This study focused on the Nile tilapia (*Oreochromis niloticus*), one of the most important aquaculture fish species globally. The aim of this study was to investigate the effects of dietary exposure to a mixture of four conventional fossil fuel-based polymers (microplastics) on the health of adult and juvenile Nile tilapia. Two experiments were conducted, with 36 juvenile tilapia (10–40 g weight) exposed for 30 days and 24 adult tilapia (600–1000 g) exposed for 7 days, the former including a natural particle (kaolin) treatment. In the adult tilapia experiment, no significant effects on intestinal health (Ussing chamber method), oxidative stress, or inflammatory pathways (enzymatic and genetic biomarkers) were observed after exposure to the microplastic mixture. However, in the juvenile tilapia experiment, significant alterations in inflammatory pathways were observed following 30 days of exposure to the microplastic mixture, indicating potential adverse effects on fish health. These results highlight the potential negative impacts of microplastics on fish health and the economics and safety of aquaculture.

## Highlights

• Nile tilapia were exposed to a fossil fuel-based microplastics mixture.
• Ingestion of microplastics causes inflammatory responses in juvenile Nile tilapia.
• Microplastics can pose a risk to the sustainable development of aquaculture.

## Graphical abstract

**Figure:**
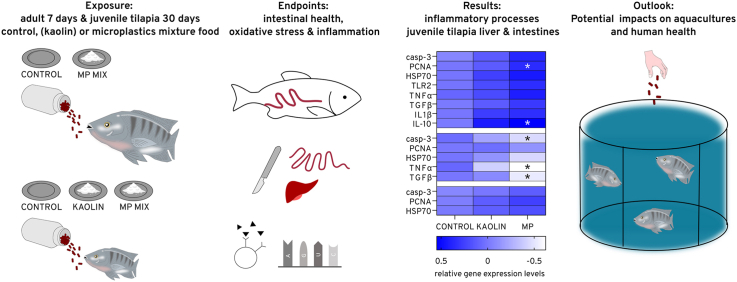

## 1 Introduction

Microplastic is an emergent pollutant of increasing concern. These synthetic particles (<5 mm [1]) are likely present in every aquatic habitat [2]. Though present in all aquatic environments, microplastic particles may be disproportionately present in inland freshwater and coastal habitats, especially in areas adjacent to anthropogenic centers [3,4]. Because of their propensity to accumulate in these habitats, microplastics represent a significant challenge to the sustainable development of aquaculture in both environments. In addition to external sources, there are many potential sources of microplastics in aquaculture environments, including the fragmentation of plastic from nets and ropes [5]. Similarly, in recirculating aquaculture systems (RAS), microplastics are likely to originate from plastic tanks, pipes, and filtration components. In addition, fish meal (a major component of fish feed) has been identified as a significant source of microplastic ingestion in farmed fish [6,7].

The uptake of microplastics in farmed fish has been conclusively demonstrated [8], through both ingestion and by translocation through the primary barriers [9]. In some cases, the presence of microplastics in farmed fish is almost double that in wild-caught fish [10]. Microplastic particles are found in the gastrointestinal tract of fish [11], but studies have shown generally low levels of microplastics accumulate in the muscle tissue of fish [12,13], suggesting a relatively low risk to human health associated with fish fillet consumption. However, sublethal effects i.e., reduced growth and fecundity, may reduce overall productivity, leading to potential concerns over food security in the future.

Exposure to microplastics can induce adverse effects in many fish species [14,15]. These effects include biochemical effects such as oxidative stress, neurotoxicity, and an imbalance in reactive oxygen species [15]. In addition, exposure to microplastics can induce physiological effects such as morphological deformities and behavioral changes in fish larvae [16,17]. The exact mechanisms for the observed effects of microplastic exposure on fish remain contentious, with effects likely dependent on many factors including particle size [18], concentration, polymer type, and chemical load of the particles (i.e., virgin vs. weathered) [19]. It is possible that an increased load of indigestible material leads to a reduction in feeding and thus to the commonly observed adverse effects on growth and fecundity, namely, food dilution [20]. Chemicals associated with microplastics [21], or the microbiome growing on the surface of the microplastics [22], may also be responsible for the observed effects. However, as the majority of microplastic effect studies do not employ the use of particle controls, it is difficult to elucidate particle effects from chemical effects in these studies. This is particularly true for fish, as a recent meta-analysis found no microplastic effect studies in fish that used natural particle controls, indicating a significant knowledge gap [23].

Nile tilapia (*Oreochromis niloticus*) was chosen as the study subject for several reasons. First, it is an important aquaculture species, being the third most farmed fish species globally [24]. Second, juvenile tilapia have previously been shown to be sensitive to microplastic exposure [25,26], which may better allow for the delineation of natural particle and microplastic effects. By characterising effects in both juveniles and adults, we provide a more complete assessment of the effects of microplastic exposure on this species. The aim of the study was to test the effects of microplastic ingestion on tilapia physiology at both juvenile and adult life stages. Sublethal endpoints investigated general life cycle parameters, oxidative stress, detoxification, and inflammatory response pathways using genetic and enzymatic biomarkers, and intestinal health via intestinal barrier and transporting functions and morphology. As the majority of effect studies on microplastics focus on a single polymer type [27], the exposure to a microplastic mixture of four conventional polymers (polyethylene terephthalate, high-density polyethylene, polypropylene, and nylon-6) which are commonly found in the environment [28,29] adds a level of environmental relevance to the present study.

## 2 Methods

### 2.1 Test particles and feed preparation

Commercial polypropylene (PP) (RIGK 1346, Borealis, Austria), polyethylene terephthalate (PET) (Steinbeis PolyVert GmbH, PET Plant AT, Völkermarkt, Austria), high-density polyethylene (HDPE) (Polyethylene Lumicene Supertough 22ST05/32ST05, Total Energies), and Nylon-6 (Sigma Aldrich, USA, CAS no. 25038-54-4) granules were ground and sieved to microplastic particles in a size range of 10–350 μm, following the protocol in König Kardgar et al. [30] with adjusted sieve sizes. Kaolin (20–35 μm) was obtained from Sigma Aldrich (CAS no 1332-58-7).

Commercial tilapia fish food (Skretting, Nutreco N·V., The Netherlands), was spiked with microplastics or kaolin particles and prepared following the protocol of König Kardgar et al. [30]. Shortly, commercial fish food was ground to powder and mixed with gelatine, red food colorant, water, and for the two spiked feeds additional particles. The prepared food was stored at −20 °C. Food was spiked with a 2 % (w/w) concentration of microplastics based on a previous study [30] (equal amounts of PP, PET, HDPE, and Nylon-6) or kaolin. The kaolin treatment was applied in experiment 1 on juvenile Tilapia and was not part of experiment 2 on adult Tilapia due to constraints in laboratory space and the limited availability of adult fish. The control feed did not contain added particles. In total, there were three different test diets in experiment 1 and two test diets in experiment 2.

### 2.2 Experimental fish and holding conditions

Male juvenile (10–40 g) and adult (600–1000 g) Nile tilapia (*Oreochromis niloticus*) were obtained from Gårdsfisk AB (Skåne, Sweden) and maintained at the Department of Biological and Environmental Sciences, University of Gothenburg. At the aquaculture farm, adult tilapia were held in full polyethylene tanks, piping, and biofilters, and juveniles were held in fiberglass tanks with polyethylene piping and biofilters (Gårdsfisk AB, Sweden). Kaolin may be used in the production of fiberglass [31]. Upon transfer to the university, the juveniles were acclimated in 120 L glass aquaria for five weeks prior to beginning the experiment. The adults were acclimated for 14 days at a stocking density of two individuals per 120 L tank. The adults were separated by an acrylic sheet. All fish were maintained at 26 ± 1 °C on a 14:10 light: dark cycle. To maintain water quality, 33 % of the water was exchanged daily. Water quality parameters, such as oxygen levels, temperature, nitrate, nitrite, and ammonia levels were regularly monitored. Tanks were siphoned after each feeding to remove leftover food and feces. During the acclimation period, juvenile fish were fed 3 % (w/w) food per fish body weight and adults ad libitum daily with commercial tilapia food pellets (Skretting, Nutreco N·V., The Netherlands). We selected 3 % of the fish's body weight for the juveniles as during the acclimation period, it was determined that this food proportion was well-accepted by the fish without significant leftover. Adult fish were fed ad libitum in the experiment as they required manual feeding with food placed directly in front of them to ensure consumption; otherwise, they exhibited reluctance to eat. In average 3–5 g of food per day were consumed by each fish. This feeding regime during the acclimation period ensured that the fish were adapted to the later experimental feeding regime.

The ethical guidelines set forth by the Swedish Board of Agriculture (Ethical permit number: 15,984–2018) were followed during the animal husbandry and feeding experiments. Daily monitoring of the fish was carried out, and throughout the experiments, all individuals remained visibly healthy based on pre-exposure observations and laboratory standards.

### 2.3 Feeding exposure

For the juvenile tilapia feeding trial, 12 fish each were randomly distributed into three 120 L aquaria (N = 36). There were three treatments: negative control (no particles present in the feed), natural particle exposure (feed spiked 2 % *w*/w 20–35 μm kaolin particles), and microplastic exposure (feed spiked with 2 % w/w 10–350 μm PP, PET, HDPE, and Nylon-6 particles). The juvenile tilapia were fed over a 30-day period from March to April 2022. The fish were fed 3 % of their body weight once per day. The experimental conditions were consistent with those of the acclimation period, including daily water changes.

For the adult feeding trial, the experiment took place in the same tanks as the acclimation. There were two treatments: 12 fish each for the negative control (no particles) and microplastic exposure (N = 24). Two fish of the same treatment shared a 120 L tank. Both feeds were the same as those used for the juvenile experiment. The adult feeding trial was carried out for a seven-day period, with the adults being fed until satiation once per day.

### 2.4 Sampling protocol

At the end of both experiments, the fish were euthanized by percussive stunning, followed by severing the spinal cord. Weight and fork length were measured before the fish were opened ventro-laterally. The weight-to-length ratio was calculated. For each fish, two proximal and distal intestine and liver samples were taken and snap-frozen in liquid nitrogen to await further gene expression analysis. Proximal intestinal tissue was sampled as the intestinal part directly following the stomach and distal intestinal tissue was taken from the part preceding the anus. For the juvenile tilapia, intestinal health was assessed using histology. For this, a 2 mm section was carefully excised and placed immediately in a 4 % buffered formaldehyde solution for histological analysis. For the adult tilapia, intestinal health was assessed using the Ussing chamber method. For this, a 4 cm section of the intestine was excised. This section was cut longitudinally to make a flat epithelium. The intestines were stored in chilled Ringer's solution (140 mM NaCl, 2.5 mM KCl, 15 mM NaHCO_3_, 1.5 mM CaCl_2_, 1 mM KH_2_PO_4_, 0.8 mM MgSO_4_, 10 mM glucose, 20 mM l-glutamine and 5 mM HEPES buffer (pH 7.8)).

### 2.5 Gene expression analysis

Liver and intestinal tissue samples were homogenized using the TissueLyzer II (Qiagen), and mRNA was extracted from N = 10 (liver, juvenile), N = 6–10 (intestine, juvenile), and N = 12 (liver, adult) fish per treatment using the RNeasy Mini Kit (Qiagen). The isolated 500 ng mRNA underwent reverse transcription into cDNA using the iScript™ cDNA Synthesis Kit (Bio-Rad Laboratories Inc.). In this study, the specific genes caspase-3 (*casp-3*), proliferating cell nuclear antigen (*PCNA*), toll-like receptor 2 (*TLR2*), tumor necrosis factor alpha (*TNFɑ*), transforming growth factor beta (*TGFβ*), interleukin 1 beta (*IL1β*), and interleukin 10 (*IL-10*) were selected as biomarkers for inflammatory response and cell apoptosis pathways. The immuno-modulatory genes were chosen based on previous research on the inflammatory responses and intestinal health in Nile tilapia after exposure to microplastics and dietary supplements [32,33]. Real-time polymerase chain reaction (RT-qPCR) was performed using the SYBR® Green Master Mix (Bio-Rad Laboratories Inc.). Each 10 μL reaction contained 20 ng cDNA and a final concentration of 500 nM forward/reverse Primer. Primer sequences and sources can be found in Supplementary dataTable A1. To ensure primer quality and monitor genomic DNA contamination, nontemplate (NTC) and no reverse transcription (NRT) controls were included for each analyzed gene. The average of three technical replicates for each individual fish for each gene was calculated. The target genes' expression levels were normalized against housekeeping genes' expression levels (beta-actin: *β-actin*, glyceraldehyde-3-phosphate dehydrogenase: *GAPDH*, and elongation factor 1-alpha: *ef-1α*), following the analysis by the 2^−ΔΔ*C*T^ method [34]. The log2 fold gene expression levels were used for data demonstration and statistical analysis. The following thermal protocol was carried out in the CFX Connect Real-Time System (Bio-Rad Laboratories Inc.): Initial denaturation (2 min at 95 °C), followed by 40 cycles of denaturation (5 s at 95 °C) and annealing (30 s at 60 °C) and concluded with a melting curve analysis (5 s at 95 °C and 5 s from 65 to 95 °C in 0.5 °C increments).

### 2.6 Enzymatic activity assays

The Cyp1a-activity in liver tissue was determined with the established ethoxyresorufin-*O*-deethylase (EROD) fluorometric assay [35] modified by Goksayr and Förlin [36]. Protein levels in both brain and liver tissue were determined with the Lowry assay, with protein standards (bovine serum albumin, BSA) [37] to normalize enzyme levels of the respective tissues. The activity of glutathione *S*-transferase (GST) was analyzed following the protocol described by Habig et al. [38], with adaptations to reading in microplates as outlined in Stephensen et al. [39]. Catalase activity was conducted based on an adapted version of the method established by Aebi [40].

### 2.7 Intestinal health in juveniles - histology

For the histological analysis, intestinal tissues were dehydrated using an ethanol gradient followed by Histolab Clear (Histolab Products AB, Askim, Sweden). After that, the samples were embedded in paraffin using a tissue processor (TP 1020, Leica, Wetzlar, Germany). Two non-consecutive cross-sections were produced for each tissue using a microtome (Shandon Scientific; Labex Instrument, Helsingborg, Sweden) and mounted on slides coated with 3′-aminopropyltriethoxysilane (APES; Merck KGaA, Darmstadt, Germany). The sections were stained using hematoxylin (Histolab Products AB, Askim, Sweden), eosin (Histolab Products AB), and alcian blue 8 GX (pH 2.5, Merck, KGaA, Darmstadt, Germany). For the morphological evaluation, three pictures of each section were taken using a 2.3 megapixel camera (PowerPack ace 2.3 MP, Basler AG, Ahrensburg, Germany) connected to a microscope (Eclipse E1000, Nikon, Tokyo, Japan, ×10 magnification). This resulted in six images per fish and intestinal region, which were analyzed for villi length, lamina propria width, submucosa width, and goblet cell count using the ImageJ software (Wayne Rasband, NIH, USA). Six fish (N = 6) were analyzed for each dietary treatment.

### 2.8 Intestinal health in adults - ussing chamber method

Barrier function and ion transport in the adult intestine were assessed using the Ussing chamber method (UCC-401; UCC-Laboratories Ltd.) described by Sundell [41], and using modifications described by Sundell and Sundh [42]. In brief, this method involves the measurement of several electrophysiological parameters from an isolated epithelium, which is bathed in a physiological saline solution (in this case, Ringer's solution for freshwater fish was used). A gas mixture consisting of 99.7 % air and 0.3 % CO_2_ provided circulation, oxygenation, and pH regulation to both chamber halves. This ensured viability of the excised intestine for the duration of the experiment. The temperature of the chambers was kept at 26 °C using a water-heated mantle.

Adaptive DC voltages were applied to the intestine every 5 min using platinum electrodes, generating currents (I) from −30 to 30 μA to prevent electrical charging. The U/I pairs were fitted to a line to determine generating currents (I). Transepithelial electrical potential (TEP) was measured using reference electrodes (REF201, red-rod, Hach-Lange). Short circuit current (SCC) was calculated as SCC = -TEP/TER, representing net active ion transport. Transepithelial resistance (TER) reflects paracellular shunt resistance, while TEP reflects net ion distribution across the epithelium due to paracellular and transcellular ion transfer.

### 2.9 Statistical analysis

Parametric data of gene expression, life cycle parameters, and histology data of juveniles were analyzed using one-way ANOVA followed by a post-hoc Tukey's multiple comparisons tests, in adults unpaired t-tests were applied. Nonparametric data of life cycle parameters, enzymatic activity, and gene expression was analyzed using Mann-Whitney tests. The intestinal permeability data was analyzed using Welch tests. All data was analyzed using GraphPad Prism version 10.0.2 for Windows, developed by GraphPad Software, LCC, San Diego, California, USA, and was used for statistical analysis in this study. A significance level of p ≤ 0.05 was considered for all statistical analyses.

## 3 Results

### 3.1 Effect on growth of juvenile tilapia

No mortality or visible signs of affected health occurred during the experiment. The length of the fish ranged between 8 and 14 cm, and weight between approximately 10 and 40 g (see Fig. A1). The individuals in the control group exhibited a weight range from 11.9 to 37.2 g (with a mean of 26.2 g), while the kaolin treatment group demonstrated weights spanning from 19.6 to 35.6 g (mean: 26.7 g), and the fish in the microplastics treatment group displayed weights ranging from 20.2 to 38.7 g (mean: 27.5 g). No life cycle parameters differed in the three treatments.

### 3.2 Effect on biomarker gene expression of juvenile tilapia

**Fig. 1:**
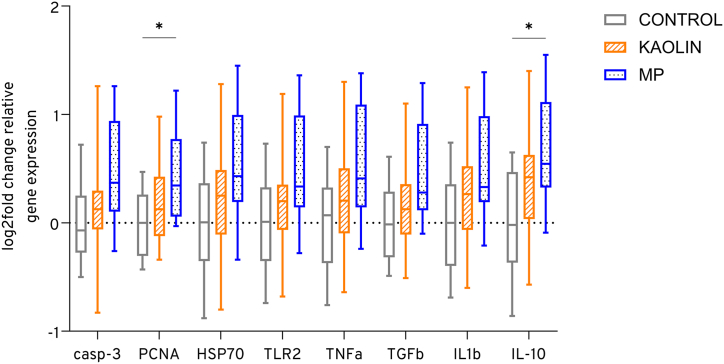
The gene expression in the liver tissue of juvenile tilapia (n = 10) was analyzed using RT-qPCR to investigate genes associated with inflammatory response and cell apoptosis following prolonged exposure to non-particle control, kaolin particles, and microplastics (MP). After 30 days of exposure, the expression levels were compared to the non-particle control group using boxplots, which show the log2-fold change. The whiskers represent the minimum and maximum values, while the median is indicated.

In all analyzed genes, we observed a trend of 1.4 times upregulation of their relative expression in the microplastic mixture treatment (mean log2fold change: 0.51 ± 0.5 SD) in liver tissue compared to control (mean: 0.00 ± 0.4 SD) and kaolin (mean: 0.21 ± 0.4 SD) treatment (Fig. 1). A progressive upregulation was observed from the control group to the kaolin treatment and further to the microplastics mixture treatment. The mean upregulation was 1.2 times in the kaolin treatment compared to the control. The expression of *PCNA* (*p*: 0.04), and *IL-10* (*p*: 0.02) was significantly upregulated (1.3 times and 1.6 times respectively) in the liver of juvenile tilapia after the 30-day ingestion of conventional microplastic mixture spiked food compared to the control. In genes without significantly different expression levels, mean differences to the control of +0.47 in *casp-3*, +0.52 in *HSP70*, +0.49 in *TLR2*, +0.54 in *TNFɑ*, +0.46 in *TGFꞵ* and +0.52 in *IL1ꞵ*.

**Fig. 2:**
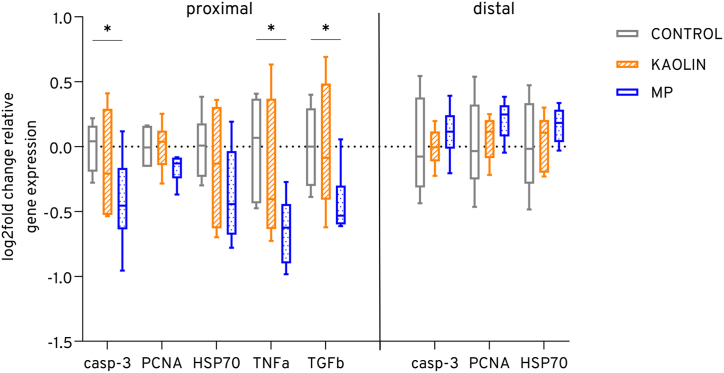
The gene expression in the proximal and distal intestinal tissue of juvenile tilapia (n = 6–10) was analyzed using RT-qPCR to investigate genes associated with inflammatory response and cell apoptosis pathways following prolonged exposure to non-particle control, kaolin particles, and microplastics (MP). After 30 days of exposure, the expression levels were compared to the non-particle control group using boxplots, which show the log2-fold change. The whiskers represent the minimum and maximum values, while the median is indicated.

Several genes were selectively studied in juvenile tilapia's proximal and distal intestinal tissue (Fig. 2). In the proximal part of the intestines, *casp-3* (*p*: 0.026), *TNFɑ* (*p*: 0.017), and *TGFꞵ* (*p*: 0.015) were significantly downregulated 0.6 to 0.7 times in the microplastic mixture exposure treatment, compared to the control. This effect was not observed in the natural particle (kaolin) treatment group. Overall, in proximal intestines, all selected genes showed a tendency of downregulation in the microplastic treatment with a mean difference of −0.40 (±0.3 SD) to the control. However, in distal intestines, a trend of gradual upregulation in kaolin (mean lof2fold change: 0.03 ± 0.2 SD) and microplastic (mean: 0.16 ± 0.15 SD) treatments compared to the control was observed, while there were no statistically significant differences.

### 3.3 Effects on intestinal tissue of juvenile tilapia

**Fig. 3:**
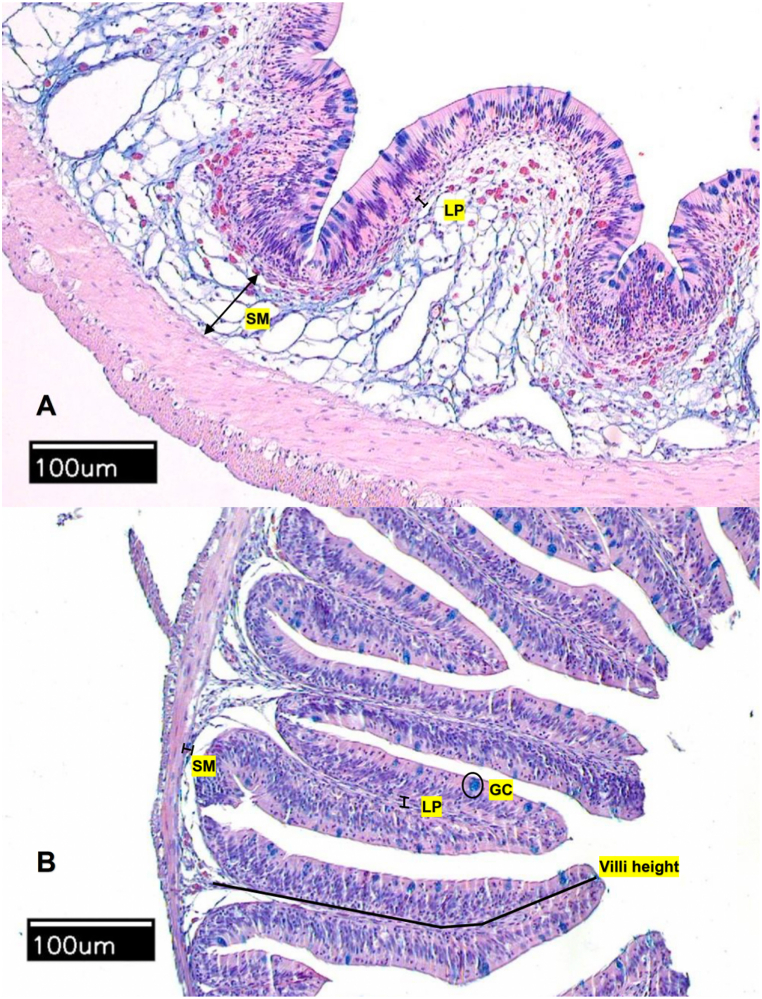
Intestinal sections from adult (A) and juvenile (B) Nile tilapia fed the control diet without added particles. SM = submucosa, LP = lamina propria, GC = goblet cell. No differences were found in the histology of intestinal sections of control, kaolin, and microplastic mixture treatments.

The histological evaluation revealed no statistical differences of kaolin or microplastic ingestion on intestinal submucosa width, lamina propria width, villi height, and goblet cell count for juvenile Nile tilapia compared to the control treatment (Table 1, Fig. 3).

**Table 1:** Histology results in intestinal tissue of juvenile tilapia after 30 days of exposure to control, kaolin, or microplastic mixture diets.

Treatment	Villi height (μm)	Lamina propria (LP) width (μm)	Submucosa (SM) width (μm)	Number of goblet cells (GC) per villi	GC/100 μm epithelium
Control	244.66 366.61 298.40 216.65 241.77 296.26	5.49 8.92 7.99 4.37 4.77 9.49	6.34 12.82 6.59 8.25 5.45 6.87	14.44 18.33 15.92 14.67 15.75 15.58	3.02 2.51 2.72 3.45 3.30 2.65
Kaolin	276.20 328.97 375.74 283.20 228.61 286.22 224.24	8.46 10.76 10.33 10.43 11.58 9.58 5.73	6.69 11.48 7.88 8.88 7.88 8.56 7.78	14.83 20.67 12.83 13.33 11.42 19.75 11.67	2.69 3.14 1.71 2.35 2.50 3.45 2.60
Microplastics	255.87 353.53 254.56 293.92 252.82 313.86	5.13 6.85 9.33 9.17 8.48 9.49	6.85 10.21 9.31 8.75 7.10 8.73	14.00 17.75 15.50 17.19 12.33 16.08	2.74 2.51 3.04 2.92 2.44 2.70

### 3.4 Effect on growth of adult tilapia

There were no instances of mortality or observable indications of impacted health of the fish throughout the experiment. Following a 7-day period of exposure to non-particle control, or microplastic mixture, the adult tilapia were measured in length and weight, and weight-to-length ratios were established. The fish's length varied between 30 and 36 cm, while their weight ranged from approximately 600 to 1000 g, with a range of 692–981 g (mean: 794 g) in the control and 640–880 g (mean: 774 g) in the microplastics treatment (see Fig. A2). Life cycle parameters of adult tilapia did not show any differences between control and microplastic treatment.

### 3.5 Effect on biomarker gene expression of adult tilapia

**Fig. 4:**
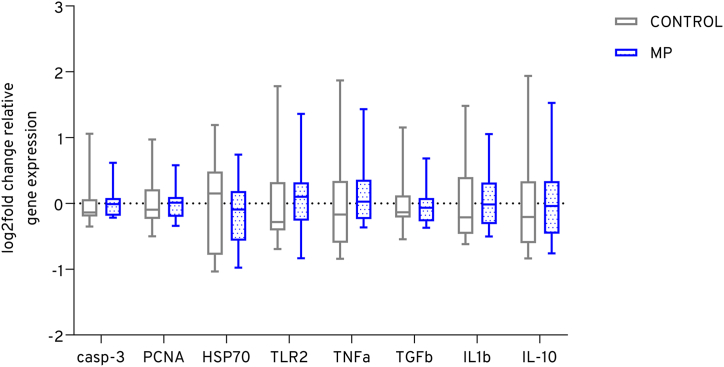
The gene expression in the liver tissue of adult tilapia (n = 12) was analyzed using RT-qPCR to investigate genes associated with inflammatory response and cell apoptosis following exposure to non-particle control and microplastics (MP). After 7 days of exposure, the expression levels were compared to the non-particle control group using boxplots, which show the log2-fold change. The whiskers represent the minimum and maximum values, while the median is indicated.

After the 7-day exposure, potential effects on the expression levels of selected genes were analyzed in the liver of adult tilapia (Fig. 4). No significant differences were observed between the control and microplastics treatment in the expression levels of inflammatory response or cell apoptosis pathway genes.

### 3.6 Effect on biomarker enzymatic activities of adult tilapia

**Fig. 5:**
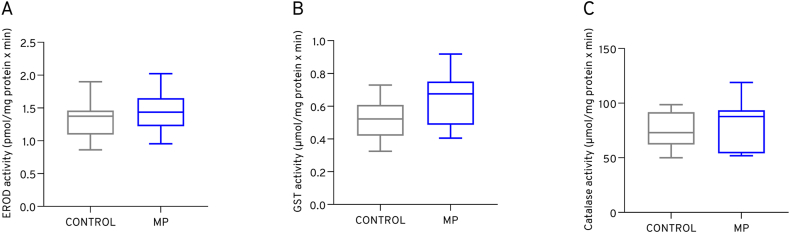
Enzymatic activity levels related to detoxification and oxidative stress measured in the liver tissue of adult tilapia after 7 days of exposure to control (no particle) or microplastic mixture (MP) food. A: ethoxyresorufin-*O*-deethylase (EROD/cyp1a) activity, B: glutathione *S*-transferase activity (GST), C: catalase activity.

No significant difference was observed in cyp1a, glutathione-*S*-transferase, and catalase activities in the liver of adult tilapia after 7-day exposure to non-particle control or microplastic mixture food (see Fig. 5).

### 3.7 Intestinal barrier function

**Fig. 6:**
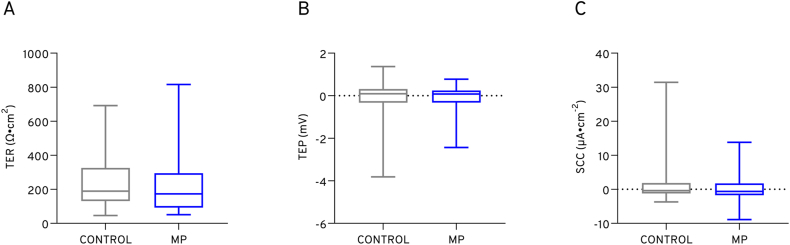
The effect of microplastic ingestion on intestinal (A) transepithelial resistance (TER), (B) transepithelial electrical potential (TEP), and (C) short circuit current (SCC) in adult tilapia (N = 24). Bars represent mean and error bars represent standard deviation.

There was no significant difference in any of the electrical parameters between the microplastic exposed and control fish (Fig. 6). For each parameter, there was a high degree of variability.

### 3.8 Effects on intestinal tissue of adult tilapia

The histological evaluation revealed no effects of microplastic ingestion on intestinal submucosa width, lamina propria width, villi length, and goblet cell count for adult Nile tilapia (Fig. 3).

## 4 Discussion

In this study, the potential effects of a microplastic mixture exposure on adult and juvenile tilapia were investigated over an acute (7-day) and a long-term (30-day) period, respectively. Our results revealed significant changes in biomarker gene expression in juvenile tilapia, indicating inflammatory responses in the liver and intestines. However, histological evaluations did not show corresponding signs of inflammation, suggesting a complex and potentially time-dependent response. The microplastics used in the present study were derived from a mixture of fossil fuel-based plastics. These included PET, PP, HDPE, and Nylon-6. As fossil fuel-based plastics remain by far the most widespread polymer types in production and in the environment to date, they are also the most prevalent forms of plastic used in aquaculture [[43], [44], [45], [46], [47], [48], [49]]. It has been shown that microplastics are present in aquaculture systems, where they likely originate from feed, clothing, and the components of the systems i.e., filtration equipment and tanks [50]. As the presence of microplastics has now been confirmed in fish aquaculture systems, it is important to understand the extent to which microplastics can harm farmed fish.

All tested genes in this study were based on the research by Standen et al. [32] and have been widely applied as biomarkers for immuno-modulatory processes following the dietary supplementation of microplastics, probiotics, or other compounds in Nile tilapia and other fish species (e.g. Refs. [32,33,51,52]). In the study by Standen et al. [32], *casp-3*, *PCNA*, *HSP70*, *TLR2*, *TNFɑ*, *TGFβ*, *IL-1β*, and *IL-10* relative expression levels were analyzed in intestines, where several gene expression levels were increased after administration of specific probiotics while no indications for inflammation were found in histology [32]. Our dietary exposure to microplastics showed similar effects on gene expression levels, while histological analysis showed no signs of inflammation in intestinal tissue. Studying the intestinal health of aquaculture fish species, which are commonly farmed in high stocking densities and varying water quality, is especially important. This is because the intestinal tract represents one of the most important interaction locations with pathogens or chemical contaminants [32,53]. Standen et al. [32] suggest that adaptive regulation of relevant genetic pathways might be beneficial for aquaculture fish.

In the juvenile tilapia, there were clear indications of inflammatory processes in the liver and intestines. Several genes related to an inflammatory response and cell apoptosis were significantly upregulated in the liver and downregulated in the proximal intestine in the microplastic mixture treatment compared to the non-particle control. Other tested genes of these pathways in the liver and intestines followed the same expression level patterns in a nearly significant manner. Though not significant, there was a gradual upregulation in the particle control (kaolin) exposed group. The significant upregulation of *PCNA*, and *IL-10* in the liver, and the downregulation of *casp-3*, *TNFα*, and *TGFβ* in intestinal tissue could be indicative of inflammatory responses and potential liver and intestinal damage due to the changes of expression levels in relevant genetic pathways. It is important to note that no evidence of inflammation was observed in the intestine of juvenile tilapia based on histological analysis. However, is it possible that a longer exposure duration may increase the likelihood of observing these more downstream effects.

While *casp-3* is important in the apoptotic pathway when the cell goes through controlled cell death, *PCNA* is a protein that is important for cell proliferation, DNA synthesis, and repair [54]. Thus, the findings of significantly increased expression of *PCNA* in the liver and a clear tendency for upregulation of *casp-3* indicate that the liver tissue is under stress caused by the microplastics, which may result in increased apoptosis followed by an upregulation in cell proliferation. Microplastics have previously been reported to induce changes in apoptotic and cell proliferating pathways [55,56]. *TLR2*, *IL1β*, and *TNFɑ* are involved in the recognition of pathogens and result in the release of pro-inflammatory cytokines [57]. Therefore, the significant downregulation of *TNFɑ* in the proximal intestines indicates a decrease in the production or activity of this inflammatory cytokine in the gastrointestinal tract, while in the liver a tendency of upregulation of *TLR2* and *IL1β* could be a sign of an increased pro-inflammatory response to the present microplastics. Both effects demonstrate a potential disturbance of this pathway in the fish's immune system.

On the other hand, *TGFβ* and *IL-10* are anti-inflammatory, and their role is to dampen the inflammatory response [58]. In the liver of juvenile tilapia both genes were significantly upregulated, while *TGFβ* was significantly down-regulated in the proximal intestine. This indicates a complex response to the microplastic exposure that involves both local and systemic immune reactions in the liver and intestines. In the liver, upregulation of *TGFβ* could show an attempt to modulate the immune response and promote tissue healing in response to potential damage caused by microplastic exposure [59,60]. The down-regulation of *TGFβ* in the proximal intestine suggests a different response compared to the liver. The down-regulation indicates a compromised barrier function and immune dysregulation of the intestinal tissue [61]. The upregulated *IL-*10 might indicate the suppression of inflammation caused by microplastic exposure and the prevention of further tissue damage in the liver [62,63].

Especially the expression of interleukins has been analyzed in the context of microplastic exposure in fish in multiple studies. Here, results are not consistent and appear to highly depend on exposure conditions, test species, and microplastic properties. While upregulation of interleukins consistent with our results was observed in zebrafish (*Danio rerio*) after exposure to nanoplastics [64], other research did not find any significant differences in interleukin levels after microplastic exposures in fish or human cell lines [51,65,66].

In juvenile tilapia, we observed differences between levels of expression of *casp-3*, *PCNA*, and *HSP70* in proximal and distal intestinal tissues. In the distal intestine, a similar upregulatory response as in the liver occurred, while in the proximal intestine, a contrary pattern of downregulation in the microplastic-exposed fish occurred. Del Piano et al. [52] also observed tissue-dependent down-regulation/upregulation of *IL-1β*, *IL-10*, *TNFɑ*, and *TGFβ*, *TLR2* in the proximal and distal intestine of gilthead seabreams (*Sparus aurata*) following ingestion of polystyrene microplastics. While *TNFɑ*, *TGFβ*, and *TLR2* were significantly upregulated in proximal intestinal tissue after the PS exposure, there was no effect on the expression levels of these genes in distal tissue. *IL-10* however was upregulated in proximal and downregulated in distal intestines of the *P*S-exposed gilthead seabreams. The expression of *IL-1β* was upregulated in both proximal and distal intestines [52]. The same group previously found these differences in proximal and distal intestinal tissue along with effects in the histology of the intestines, as a reduced villi height, reduced number of goblet cells, and elevated signs of oxidative stress by ingestion of polystyrene microplastics in seabream [67].

We suppose that down-regulated levels of inflammatory genes in the proximal intestines occur as an immediate response to the microplastics' presence as a form of damage to the intestinal tissue, while an upregulation in the distal areas of the intestines could represent a later response to the pollutant.

A down-regulation of *casp-3*, *PCNA*, and *HSP70* in proximal intestinal tissue in this experiment indicates a reduced apoptotic response, decreased cell proliferation, and compromised cellular stress response, potentially impacting cellular health and tissue function. On the other hand, upregulation of these genes in the distal intestines may indicate an enhanced apoptotic response, increased cell proliferation for repair, and an improved cellular stress response, a process to mitigate the effects of the microplastics and restore normal cellular functions [[68], [69], [70], [71]].

Ahmadifar et al. [33] conducted a study comparative to ours using Nile tilapia fingerlings which were exposed to polyethylene microplastics for 28 days. In line with our results, life cycle parameters such as growth were not affected by microplastic treatments. However, long-term ingestion of polyethylene microplastics led to an increase in activity levels of antioxidant enzymes, including catalase and glutathione peroxidase in the liver [33]. Ahmadifar et al. [33] also found signs of hepatic inflammatory responses and detoxification processes on a molecular level in the juvenile tilapia after ingestion of microplastics. In accordance with their findings, we observed up-regulation of interleukins, *TNFα*, and *cyp 450*.

In our second experiment on adult, market-size tilapia we did not find any effects impacting intestinal health or inflammatory pathways after the ingestion of microplastics for 7 days. The intestinal barrier function did not differ from the control treatment. This lack of impact was also evident in histology analyses which did not show any differences between the two treatments on intestinal status, which was similar to the result of histology analyses of juveniles with relatively short exposure durations as well.

Deviating from our results, Hamed et al. [26] found clear histological alterations in the cellular and tissue level of the kidneys, liver, muscle, gills, pancreas, spine, and intestines of juvenile Nile tilapia after 15 days of exposure to microplastics in a dose-dependent manner. In their study, Nile tilapia were exposed to concentrations ranging from 1 to 100 mg/L microplastics in water in a size below 100 nm [26]. With our experimental set-up, though including different particle sizes, exposure duration, and exposure scenarios (water versus food), we could not reproduce these histological effects in juvenile Nile tilapia.

Polyethylene and fiberglass were the material of tanks and pipes in the aquaculture where we received our experimental tilapia (Gårdfisk AB, Sweden), and kaolin may be used in the production of fiberglass. The inclusion of natural particles like kaolin in toxicity studies is important to differentiate between particle effects versus chemical toxicity, though these exposures are often lacking in microplastic studies [72]. Chronic ingestion of 2 % w/w kaolin particles in fish food has been shown to cause oxidative stress and endocrine disruption in the liver of juvenile perch (*Perca fluviatilis*) [30]. While we did not find any significant impact of kaolin ingestion in tilapia, we observed a gradual upregulation of several genes from kaolin to the microplastic mixture treatment compared to the control. There was no significant difference between kaolin and microplastic treatments. This could indicate that kaolin as a particle leads to insignificant effects, which are then increased in the microplastic-exposed fish due to the chemical properties of the polymers, in addition to their particulate nature. Our study was conducted on virgin plastic pellets, milled to microplastics. While virgin polymers do not contain many additives, plastics will contain processing agents and unreacted monomers [73], and the occurrence of by-products from the manufacturing process such as specific oligomers cannot be ruled out [74]. It has been previously shown that microplastics claimed as ‘virgin’ do contain a variety of chemical compounds such as decanoic acids, or common emulsifiers and plasticizers [75]. However, the polymers and their specific structures in our study probably led to significant signs of inflammation compared to the control. We conclude that a mixture of conventional, relevant plastic particles showed increased hazards over natural particles in fish.

The Nile tilapia is one of the most important farmed fish worldwide and an important food source for humans [76]. Microplastics are found in both wild and farmed fish [77]. Aquacultures are a main source of protein for human consumption but the microplastic accumulation in fish poses a risk for humans to be exposed to the ingestion of microplastics as well [77]. Therefore, this issue poses two potential risks: microplastic accumulation in Nile tilapia could result in trophic transfer to humans adding to the exposure burden and potential health impacts [78,79], while simultaneously having adverse effects on tilapia health. This can result in a decrease in fillet quality and could cause serious economic issues for aquacultures [80]. It was shown that microplastic exposure can result in a loss in redness of trout fish fillets and thus the quality can be compromised [75]. Wu et al. [80] summarized the risks for aquacultures posed by microplastic contamination including oxidative stress, disruption in growth, reproduction, and mortality of the fish resulting in detriments in the economy of aquacultures and adverse effects on human health by consumption of aquaculture products. With the results of our study, we can confirm that exposure of one of the most important aquaculture fish, the Nile tilapia, to fossil fuel-based microplastics, is associated with relevant negative effects on their health and that a microplastic reduction in aquacultures should therefore not be neglected to ensure a stable economic status.

## 5 Conclusion

This study investigated the impact of exposure to a mixture of microplastics on adult and juvenile Nile tilapia over different time periods. In juveniles, significant gene expression changes suggested potential inflammatory processes in the liver and intestines, with distinct responses in proximal and distal intestines. However, short-term exposure in adults resulted in no discernible effects on intestinal health or inflammatory pathways. The study employed microplastics from fossil fuel-based plastics which are commonly used in aquaculture, emphasizing their potential harm to fish. Notably, the study's main gap lies in the absence of varying concentrations of microplastics, different exposure periods, and general variability in other experimental settings for future research. The study underscores the need to address microplastic pollution in aquaculture for fish health, economic stability, and consumer safety, pointing to a critical area for future research and intervention.

## Data availability

The datasets generated and analyzed during the current study are available in the Zenodo repository, 10.5281/zenodo.8,385,419.

## Ethics statement

The ethical guidelines set forth by the Swedish Board of Agriculture (Ethical permit number: 15,984–2018) were followed during the animal husbandry and feeding experiments.

## Funding

This project has received funding from the European Union's 10.13039/501100007601Horizon 2020 research and innovation programme under the Marie Skłodowska-Curie grant agreement No 860720. Additional funding was received from the Helge Ax:son Johnsson Foundation under No F23-0361 and the 10.13039/501100004359Swedish Research Council for Sustainable Development FORMAS 2018–01201.

## Author contributions

**Azora König Kardgar:** Conceptualization, Data curation, Formal analysis, Investigation, Methodology, Project administration, Visualization, Writing – original draft, Writing – review & editing. **Darragh Doyle:** Conceptualization, Data curation, Formal analysis, Funding acquisition, Investigation, Methodology, Project administration, Visualization, Writing – original draft, Writing – review & editing. **Niklas Warwas:** Data curation, Formal analysis, Investigation, Methodology, Writing – original draft, Writing – review & editing. **Terese Hjelleset:** Data curation, Formal analysis. **Henrik Sundh:** Conceptualization, Project administration, Resources, Supervision, Writing – review & editing. **Bethanie Carney Almroth:** Conceptualization, Funding acquisition, Project administration, Resources, Supervision, Writing – review & editing.

## Declaration of competing interest

The authors declare that they have no known competing financial interests or personal relationships that could have appeared to influence the work reported in this paper.

## List of abbreviations

APES: 3′-aminopropyltriethoxysilane
BSA: bovine serum albumin
CaCl_2_: calcium chloride
casp 3: caspase 3
cDNA: complementary DNA
CO_2_: carbon dioxide
Cyp1a: cytochrome P450
DC: direct current
ef-1α: elongation factor 1-alpha
EROD: ethoxyresorufin-*O*-deethylase
GAPDH: glyceraldehyde-3-phosphate dehydrogenase
GC: goblet cell
GST: glutathione *S*-transferase
HDPE: high-density polyethylene
HEPES: 4-(2-hydroxyethyl)-1-piperazineethanesulfonic acid
I: electric current
IL-10: interleukin 10
IL1β: interleukin 1 beta
KCl: potassium chloride
KH_2_PO_4_: potassium dihydrogen phosphate
log2: binary logarithm
LP: lamina propria
MgSO_4_: magnesium sulfate
mM: millimolar
MP: microplastics
mRNA: messenger ribonucleic acid
NaCl: sodium chloride
NaHCO_3_: sodium bicarbonate
nM: nanomolar
NRT: no reverse transcription
NTC: nontemplate control
PCNA: proliferating cell nuclear antigen
PET: polyethylene terephthalate
PP: polypropylene
RAS: Recirculatory Aquaculture System
RT-qPCR: real-time polymerase chain reaction
SCC: short circuit current
SM: submucosa
TEP: transepithelial electrical potential
TER: transepithelial resistance
TGFβ: transforming growth factor beta
TLR2: toll-like receptor 2
TNFɑ: tumor necrosis factor alpha
U: voltage
β-actin: beta-actin
μA: micro ampere
