# Supplementary material for: Microplastics in aquaculture - Potential impacts on inflammatory processes in Nile tilapia
Source: Heliyon. 2024 Apr 30;10(9):e30403. doi: 10.1016/j.heliyon.2024.e30403 (PMC11079099; doi:10.1016/j.heliyon.2024.e30403)
Supplement: Multimedia component 1 [file mmc1.docx]

**Microplastics in aquaculture - potential impacts on inflammatory processes in Nile tilapia**

Azora König Kardgar¹, Darragh Doyle¹, Niklas Warwas¹, Terese Hjelleset¹, Henrik Sundh¹, Bethanie Carney Almroth¹

¹Department of Biological and Environmental Sciences, University of Gothenburg, Gothenburg, Sweden

Azora König Kardgar; azora.konig@bioenv.gu.se ; https://orcid.org/0000-0003-2800-2342

Darragh Doyle; darragh.doyle@bioenv.gu.se ; https://orcid.org/0000-0003-4055-035X

Niklas Warwas; niklas.warwas@bioenv.gu.se ; https://orcid.org/0000-0002-5217-4294

Terese Hjelleset; gushjete@student.gu.se ; https://orcid.org/0009-0007-2937-5200

Henrik Sundh; henrik.sundh@bioenv.gu.se ; https://orcid.org/0000-0002-1459-5450

Bethanie Carney Almroth; bethanie.carney@bioenv.gu.se ; https://orcid.org/0000-0002-5037-4612

**Supplementary data**

**
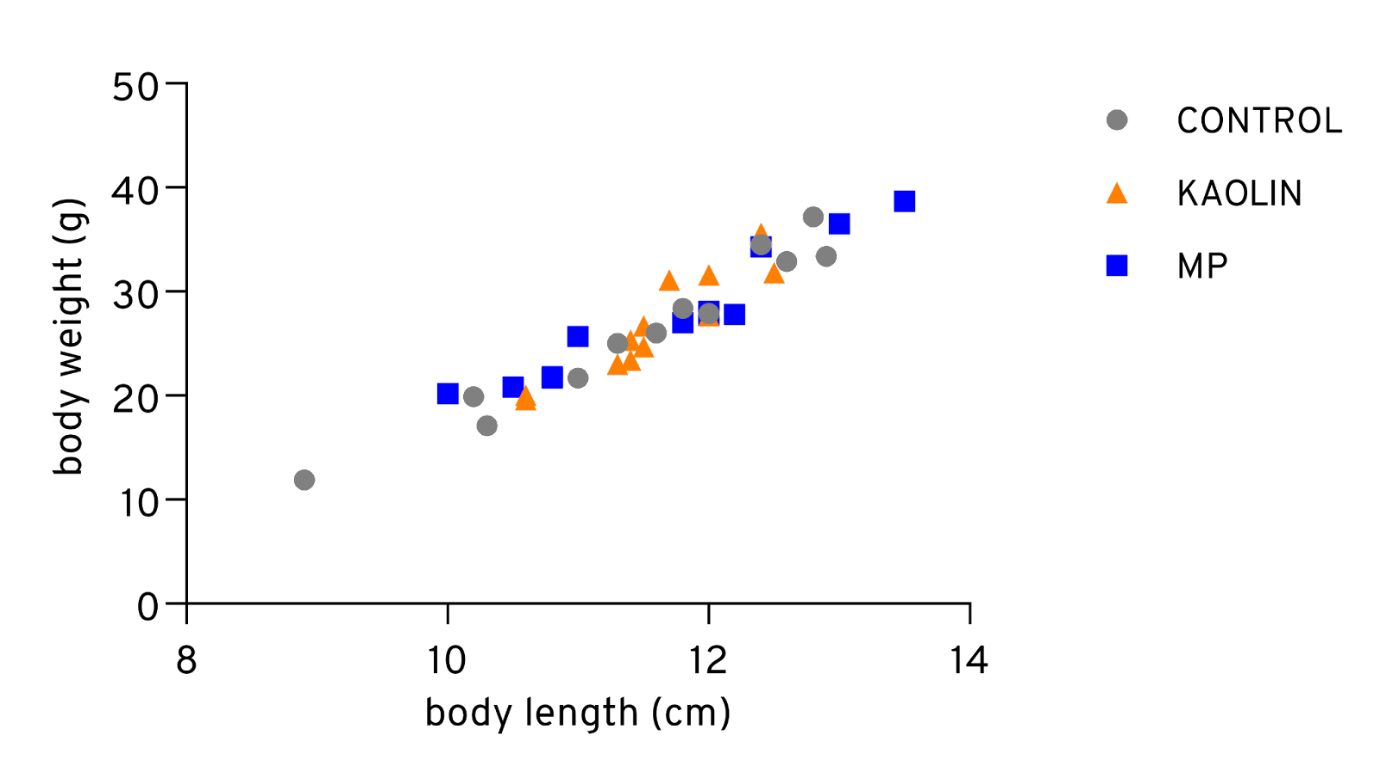
**

Figure A1: Representation of body weight to body length of juvenile tilapia after 30 days of exposure to either control (no particles), kaolin, or microplastic mixture (MP) spiked food.

*.*

**
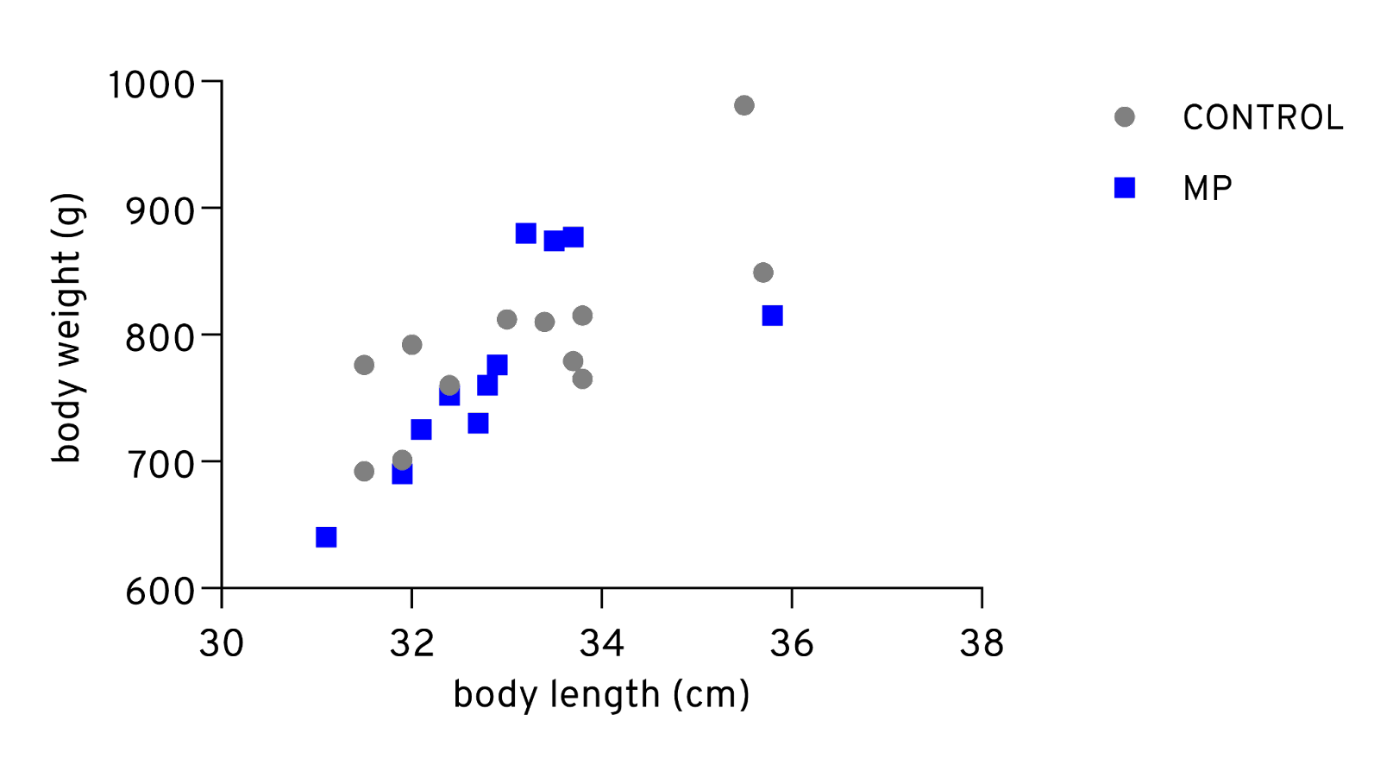
**

Figure A2: Representation of body weight to body length of adult tilapia after 7 days of exposure to control (no particles) or microplastic mixture (MP) spiked food.

Table A1: Gene expression qPCR Primer sequences *Oreochromis niloticus.*

| Gene | Forward (fw) sequence (5’-> 3’) | Reverse (rev) sequence (5’-> 3’) | Tm (°C) fw/rev | MW (g/mol) fw/rev | Amplicon size (bp) fw/rev | NCBI ID |
| --- | --- | --- | --- | --- | --- | --- |
| b-actin | TGACCTCACAGACTACCTCATG | TGATGTCACGCACGATTTCC | 60.3/ 57.3 | 6639.32/ 6067.94 | 22/ 20 | KJ126772.1 |
| gapdh | CCGATGTGTCAGTGGTGGAT | GCCTTCTTGACGGCTTCCTT | 59.4/ 59.4 | 6204.03/ 6025.87 | 20/ 20 | JN381952.1 |
| ef-1α | TGATCTACAAGTGCGGAGGAA | GGAGCCCTTTCCCATCTCA | 57.9/ 58.8 | 6519.28/ 5699.69 | 21/ 19 | AB075952.1 |
| casp-3 | GGCTCTTCGTCTGCTTCTGT | GGGAAATCGAGGCGGTATCT | 59.4/ 59.4 | 6056.88/ 6222.07 | 20/ 20 | GQ421464.1 |
| PCNA | CCCTGGTGGTGGAGTACAAG | AGAAGCCTCCTCATCGATCTTC | 61.4/ 60.3 | 6198.04/ 6630.3 | 20/ 22 | XM_003451046.2 |
| HSP70 | ACCCAGACCTTCACCACCTA | GTCCTTGGTCATGGCTCTCT | 59.4/ 59.4 | 5950.88/ 6065.9 | 20/ 20 | FJ213839.1 |
| TLR2 | GCAGTGCCTTGAGTCTTGATC | ACCGTGGAGATCGAGAACCT | 59.8/ 59.4 | 6428.16/ 6151.03 | 21/ 20 | XM_005460165 |
| TNFα | CCAGAAGCACTAAAGGCGAAGA | CCTTGGCTTTGCTGCTGATC | 60.3/ 59.4 | 6795.49/ 6065.9 | 22/ 20 | AY428948.1 |
| TGFβ | GTTTGAACTTCGGCGGTACTG | TCCTGCTCATAGTCCCAGAGA | 59.8/ 59.8 | 6468.19/ 6366.14 | 21/ 21 | XM_003459454.2 |
| IL1β | TGGTGACTCTCCTGGTCTGA | GCACAACTTTATCGGCTTCCA | 59.4/ 57.9 | 6114.95/ 6341.12 | 20/ 21 | XM_005457887.1 |
| IL-10 | CTGCTAGATCAGTCCGTCGAA | GCAGAACCGTGTCCAGGTAA | 59.8/ 59.4 | 6406.17/ 6151.03 | 21/ 20 | XM_003441366.2 |
